# A novel approach to radioembolization treatment planning using a hybrid [^99m^Tc]Tc-MAA contrast enhanced SPECT/CT

**DOI:** 10.1007/s00259-026-08027-6

**Published:** 2026-07-11

**Authors:** Grace Keane, Marnix. G. E. H. Lam, Maarten. L. J. Smits, Guus. M. Bol, Hugo. W. A. M. de Jong, Rutger. C. G Bruijnen, Arthur. J. A. T. Braat

**Affiliations:** 1 Department of Radiology and Nuclear Medicine, University Medical Center Utrecht, Utrecht, The Netherlands; 2 Boston Scientific Corporation, Marlborough, MA USA; 3 Department of Nuclear Medicine, Netherlands Cancer Institute, Amsterdam, The Netherlands

**Keywords:** Technetium-99m, Macroaggregated-albumin (MAA), imaging, segmentation, SPECT/CT, contrast-enhanced CT, Yttrium-90

## Abstract

**Purpose:**

Dosimetry-guided treatment planning is central to radioembolization. Guidelines recommend segmenting volumes on baseline contrast-enhanced CT (ceCT) and registering to a SPECT/low-dose CT. However, this conventional approach is vulnerable to spatial misregistration and potential disease progression. This study evaluates a hybrid SPECT/ceCT-based planning methodology, hypothesising that it yields significantly different dosimetry metrics compared to the conventional workflow.

**Materials and methods:**

Hybrid SPECT/ceCT was performed in 24 patients with hepatocellular carcinoma. Treatment planning followed both the conventional and hybrid workflow, where delineation was performed directly on SPECT/ceCT. Quantification of dosimetric and positional differences between volumes from the two workflows was performed using Simplicit^90^Y (Mirada Medical) and SurePlan Liver Y90 (MIM) software, respectively. Outcomes were evaluated by Bland-Altman analysis and contour intercomparison indices.

**Results:**

Direct delineation on SPECT/ceCT yielded higher index lesion (largest single tumour) doses (6% increased) (mean: 19.5 Gy, 95% limits of agreement [LoA]: -43.9 Gy, 83.0 Gy) and lower normal tissue doses (11% decrease) (mean: -17.6 Gy 95% LoA: -104.4 Gy, 69.3 Gy). Index lesion volume increase between baseline CT and SPECT/ceCT was 14% (95% LoA: -55.9%, 82.9%). Index lesion dose (*p* = 0.01) and volume (*p* = 0.01) differed significantly between workflows. The median time interval between baseline imaging and SPECT/ceCT was 6 weeks (IQR: 3.7 to 9.3 weeks). The median deviation of the index lesion between the baseline diagnostic image and hybrid SPECT/ceCT image was 4.9 ± 2.2 mm and there was an average DICE score of 0.6 ± 0.2.

**Conclusion:**

Hybrid [99mTc]Tc-MAA SPECT/ceCT treatment planning is technically feasible and leads to significant differences in dosimetry metrics compared with the conventional method.

**Supplementary Information:**

The online version contains supplementary material available at https://doi.org/10.1007/s00259-026-08027-6.

## Introduction

Radioembolization using yttrium-90 (^90^Y) glass microspheres (TheraSphereTM, Boston Scientific Corporation, Marlborough, MA, USA) is an established locoregional therapy for hepatocellular carcinoma (HCC) [1]. Evidence increasingly demonstrates that the efficacy of radioembolization is maximised when personalized dosimetry is employed during treatment planning [2–4]. Personalised dosimetry involves analysing the distribution of intra-arterially injected technetium-99m (^99m^Tc) macroaggregated albumin (MAA) using pre-procedural SPECT/CT scans. The activity accumulation within tumour, normal liver, and lung tissue informs absorbed dose calculations through a multi-compartment dosimetry (MCD) analysis, based on the medical internal radiation dose (MIRD) schema [5, 6]. This approach allows evaluation of mean absorbed dose for each compartment, and consequently the selection of a prescribed activity that maximizes tumour dose while minimizing toxicity in non-target tissue.

Current guidelines recommend volumes be segmented on baseline diagnostic imaging consisting of multi-phase contrast enhanced CT (ceCT) or MRI and registered to the separately acquired SPECT/CT, with a non-contrast enhanced low dose CT (ldCT) [7]. Uncertainty in spatial alignment is inherent in the current treatment planning approach as registration of the baseline diagnostic imaging and corresponding structure set with the SPECT/ldCT is necessary. Scanning position at the two time points is not matched, which leads to misregistration. Consequently, inaccurate volume of interest (VOI) segmentation (using the separate ceCT as a basis) may result, degrading [^99m^Tc]Tc-MAA SPECT quantitation for the purpose of dosimetry [8].

Additionally, if a substantial time interval elapses between diagnostic imaging and SPECT/ldCT interval progression becomes a potentially significant variable, especially for patients with rapidly progressive disease. Although guidelines recommend a maximum interval of 30 days [7], intervals of 60–90 days are reported in literature [3, 9]. Generally, minimising this time interval is preferable to reduce the risk of tumour progression.

In short, the delineation of VOIs is a crucial planning step, with inaccurate segmentation leading to inaccurate activity prescription and a potential loss of treatment efficacy or increased risk of toxicity. Strategies to minimise registration errors and limit the opportunity for interval progression are required. One way to achieve this, is to perform a hybrid [^99m^Tc]Tc-MAA SPECT/contrast enhanced CT (SPECT/ceCT). A diagnostic quality, ceCT acquired in the same frame of reference as the [^99m^Tc]Tc-MAA SPECT, and therefore implicitly registered, may be valuable as no error is introduced from registration and/or interval progression in the dosimetry analysis. Although multiple processes (attenuation, scatter within the patient and detector, finite system resolution (partial volume), image noise, and smoothing filters) [10–13], together with variables within the dose calculation (operator variability [14, 15] and imperfections in [^99m^Tc]Tc-MAA as a surrogate for ^90^Y [16–19]) introduce uncertainty in dosimetry measurements, eliminating registration error and interval progression constitutes a meaningful step towards improved accuracy.

This study evaluates whether hybrid SPECT/ceCT-based treatment planning yields significantly different dosimetry metrics compared to the conventional pre-treatment radioembolization planning approach, which relies on segmentation using baseline imaging and registration with SPECT/ldCT.

## Materials and methods

### Study design and inclusion/exclusion criteria

A single-centre, retrospective study of patients treated with ^90^Y glass microspheres for HCC was conducted. Subjects were eligible for this study if they had confirmed hepatocellular carcinoma (on imaging or histopathology), with up to three well-defined unilobar tumours and at least one measurable lesion ≥ 2.5 cm (to minimise the impact of partial volume effects [20]). Main exclusion criteria included contraindication to intravenous contrast administration (renal insufficiency i.e. eGFR < 30 ml/min/1.73 m^2^, or contrast agent allergy), contraindications to radioembolization as assessed by [^99m^Tc]Tc-MAA scintigraphy (including high pulmonary shunting leading to a lung dose > 30 Gy or digestive shunting uncorrectable by coil embolization) and unavailability of baseline imaging (CT/MRI) or [^99m^Tc]Tc-MAA SPECT/ceCT of sufficient quality. If patients received multiple [^99m^Tc]Tc-MAA administrations in the same pre-treatment angiography study (i.e. multiple injection positions) they were also excluded.

The study analysis consisted of two parts: firstly, the calculation of volume and mean absorbed doses to tumour and normal tissue using segmentation derived from the baseline diagnostic images versus segmentation derived from [^99m^Tc]Tc-MAA SPECT/ceCT, and intercomparisons. Secondly, evaluation of morphological differences between segmentations created via the two workflows, quantified using validated indices, including the Hausdorf distance, mean distance to agreement and DICE similarity coefficient [21].

Pre-treatment scout procedure images were acquired using a Symbia T16 (16 slice) SPECT/CT (Siemens, Erlangen), 30 min post injection of approximately 150 MBq [^99m^Tc]Tc-MAA. A circular orbit using low-energy high-resolution collimators was used for acquisition, with a 128 × 128 matrix. Reconstruction was via ordered-subset expectation maximisation (OSEM) iterative reconstruction with Flash3D (6 subsets, 8 iterations). Image corrections included attenuation correction (ldCT-based), scatter correction (dual energy window) and partial volume correction (point spread function modelling); a post-reconstruction 5 mm Gaussian filter was also applied. A low dose CT (110kVp, 40mAs) was first acquired, followed by a ceCT (110kVp, 150mAs) in which 125 mL of Omnipaque contrast agent was administered at 4 mL/s and scanning commenced 20 s post bolus in order to acquire the late arterial phase. Both these CT acquisition were acquired as part of routine clinical care. Patient records were audited to identify any instances of contrast-induced nephropathy following contrast administration.

### Dosimetry analysis

Patients were analysed in Simplict^90^Y (Mirada medical) where the whole liver, perfused volume, index lesion (single largest lesion), perfused normal tissue and whole liver normal tissue were segmented using the baseline anatomical imaging (conventional workflow) and [^99m^Tc]Tc-MAA SPECT/ceCT (hybrid workflow) respectively, resulting in a total of two dosimetry sessions per patient. The VOIs were delineated on the SPECT/ceCT dataset (hybrid workflow) during patient treatment, where personalised dosimetry was utilised to guide activity selection. Retrospectively VOIs were recreated on the baseline CT (conventional workflow) in an independent analysis, by a nuclear medicine physician blinded to the prior segmentations. All patients were treated according to partition modelling, with a target mean tumour absorbed index lesion dose of at least 205 Gy and whole healthy liver absorbed dose < 75 Gy. Use of the conventional method or the CeCT method was upon treating physicians discretion.

For the hybrid SPECT/ceCT workflow, segmentations were created directly on the SPECT/ceCT. The whole liver volume was created using an automatic segmentation tool based on a contrast-edge detection algorithm, the perfused volume and index lesion were manually segmented by a nuclear medicine physician (Arthur Braat) and finally a Boolean operator was used to create the perfused normal tissue and whole liver normal tissue. The lung shunt fraction (LSF) value was entered (based on a geometric mean derived from planar scintigraphy) as was the injected activity and residual fraction. The multi-compartment MIRD calculation method was selected and by application of a patient relative calibration and local deposition model, together with the corrected activity and volumes of interest, dose to each of the compartments was calculated via Eq. 1:

$$\:{D}_{VOI}=\:\frac{(50\:\times\:\:{A}_{liver})}{({M}_{VOI}\:\times\:\:{\sum\:C}_{i})}{\:C}_{VOI}$$

Where D_VOI_ is the dose for a given volume of interest, the factor 50 (J/GBq) is the energy deposited in the region over time per activity injected for ^90^Y, A_liver_ is the injected activity after accounting for the residual and lung fraction, M_VOI_ is the mass of the VOI, $\:\sum\:{C}_{i}$ is the total counts in the perfused volume and C_VOI_ is the counts in the VOI.

For the conventional workflow, registration of the baseline diagnostic dataset and the SPECT/ldCT was performed. Automatic rigid registration was applied as standard and was refined via manual adaptation, a visual assessment of the result was performed using semi-transparent overlays. Registered images were used to assist in location of structures, VOIs were recreated and dose to the respective compartments calculated as per the above. A time gap of ≥ 8 weeks elapsed between completion of the separate workflows to reduce recall bias [22].

### Morphological analysis

Secondly, the baseline CT and registered SPECT/ldCT with the corresponding structure set from the conventional workflow, and SPECT/ceCT with structure set from the hybrid workflow, were imported into SurePlan LiverY90 (MIM Software Inc). Images from the two workflows were aligned using rigid registration to create a fused dataset containing all contours. Statistical comparisons between the index lesion from the conventional and hybrid workflows were conducted using DICE, Hausdorff distance, and mean distance to agreement metrics.

### Data Collected

The dosimetry endpoints included total mean absorbed dose for whole liver (D_whole_), perfused volume (D_perf_), perfused volume normal tissue (D_n, perf_), whole liver normal tissue (D_n, whole_) and index lesion (single largest lesion = D_index_) derived via the conventional workflow and the hybrid workflow using SPECT/ceCT. The morphological endpoints included the contour intercomparison indices including Hausdorff distance (HD), mean distance to agreement (mDTA), and DICE similarity coefficient for index lesion created via the two workflows.

### Statistical analysis

All statistical tests were performed using Python (3.9.7) reported p-values are two sided with a significance level of 0.05.

Bland-Altman analysis [23] was used to evaluate the agreement between tumour dose and normal tissue dose determined via the conventional workflow and hybrid workflow. The confidence intervals represent the 95% limits of agreement. A paired t-test was used to generate p values.

The mean percentage difference was calculated for all dosimetry endpoints between the conventional and hybrid workflow and computed as follows:

$$\begin{array}{c}\:Mean\:Percentage\:Difference=\\\frac{{\sum\:}_{n=1}^{N}\frac{\left({D}_{hybrid,\:n}-{D}_{conventional,\:n}\right)}{{D}_{conventional,n}}}{N}\end{array}$$

Where D_hybrid_ is the dose determined via the hybrid SPECT/ceCT workflow, D_conventional_ is the dose determined via the conventional workflow and N is the total number of cases.

## Results

### Baseline characteristics

Patients treated with ^90^Y glass radioembolization for HCC between January 2021 and December 2023 were screened for inclusion. Of the 48 patients evaluated, 24 were included in the final analysis. Exclusions comprised 18 patients with multiple [^99m^Tc]Tc-MAA administrations during pre-treatment angiography, four patients with insufficient-quality SPECT/ceCT images, one patient with insufficient quality baseline imaging and one patient with tumour size < 2.5 cm.

The mean age of patients at the time of radioembolization was 69 ± 11 years, and 75% were male. The Barcelona Clinic Liver Cancer (BCLC) staging distribution was 50% BCLC C, 29% BCLC B, and 21% BCLC A. Child-Pugh classification was predominantly A5 (63%), followed by A6 (21%) and B7 (17%). Portal vein invasion was present in 29% of patients. A total of 24 radioembolization sessions were performed, targeting 26 lesions. Segmental treatments constituted 29% of cases, unilobar treatments 63% and bilobar treatments 8%.

The median injected activity was 1.9 GBq (IQR: 0.9 to 3.2 GBq), The median lung shunt fraction was 2.0% (IQR: 0.7 to 2.8%) and anticipated median lung dose was 3.0 Gy (IQR: 0.3 to 4.3 Gy). There were no patients that received either ≥ 30 Gy to the lungs or D_whole_ ≥150 Gy in either workflow (which is consistent with the instructions for use for glass microspheres [24]). An overview is given in Table 1.

**Table 1:** Baseline characteristics

Parameter	All Patients (N=24)
Population	
Mean Age (range) (years)	69 (44-88)
Gender	
Male	18 (75%)
Female	6 (25%)
Liver Cirrhosis	18 (75%)
ECOG Status	
0	13 (54%)
1	7 (29%)
2	4 (17%)
Tumour Characteristics	
BCLC Status	
A	5 (21%)
B	7 (29%)
C	12 (50%)
Number of Tumours	26
Tumour Volume (mL)	58.4
Location	7 (29%)
Segmental	15 (63%)
Unilobar	2 (8%)
Bilobar	
Portal Vein Invasion	7 (29%)
Liver functional reserve test	
Child Pugh Status	
A5	15 (63%)
A6	5 (21%)
B7	4 (17%)
ALBI Score	
Grade 1	9 (38%)
Grade 2	15 (63%)
Baseline Laboratory	
Bilirubin (IQR) (mg/dL)	1.2 (1.0-1.7)
Albumin (IQR) (g/dL)	3.8 (3.3-4.0)
Procedural	
Lung shunt fraction (IQR) (%)	2.0% (0.7-2.8)
Injected activity (IQR) (GBq)	1.9GBq (0.9-3.2)

**Fig. 1:**
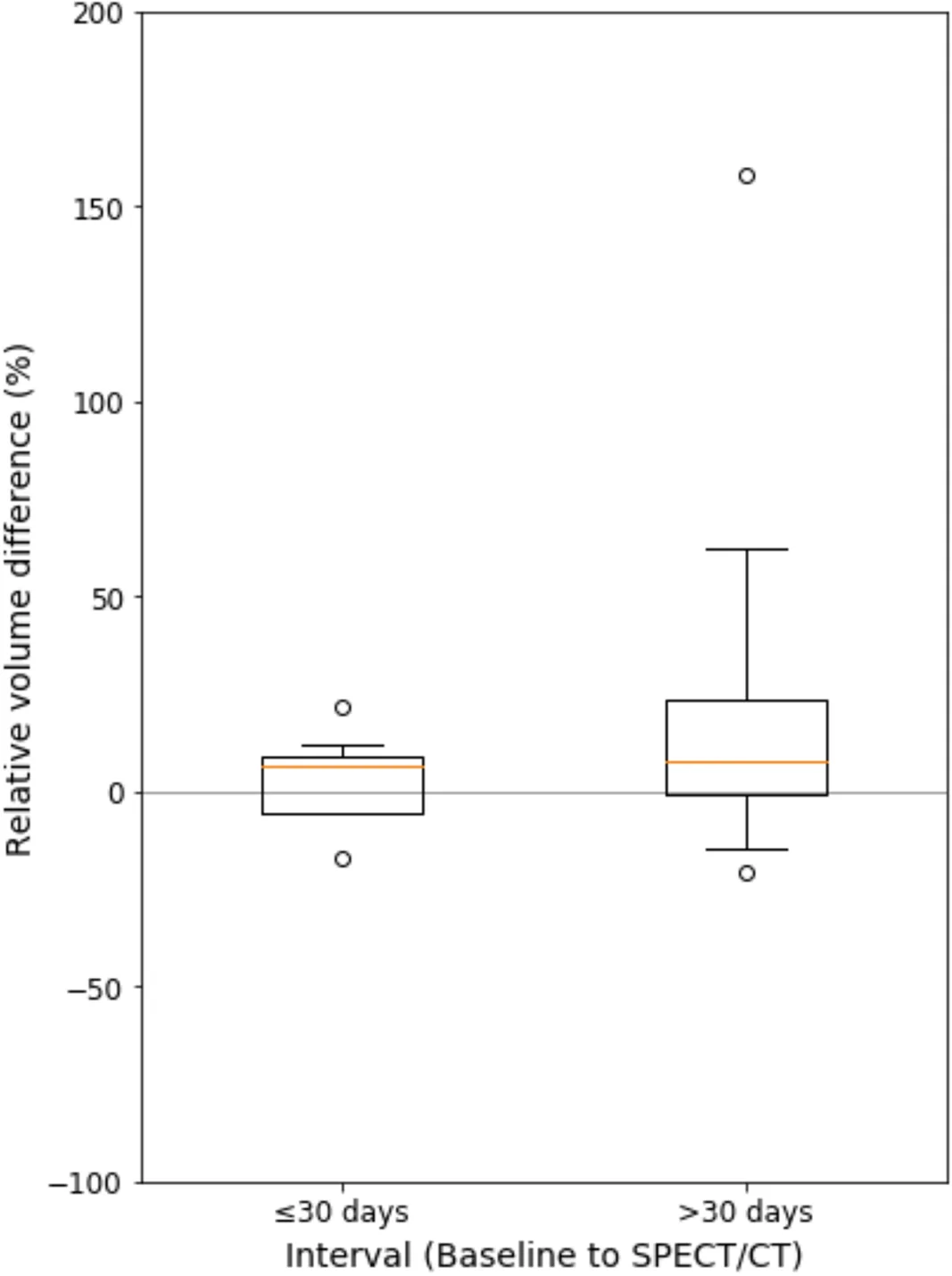
Box plot summarising the relative volume difference in index lesion for patients with a time interval between baseline imaging and SPECT/CT of < 30 days (left) and > 30 days right). The box represents the 25th to 75th percentile range and the central horizontal line the median value. The whiskers represent the 5th and 95th percentiles and the dots represent outliers

There was a median time interval of 5.9 weeks (IQR: 3.7 to 9.3 weeks) between baseline imaging (CT/MRI) and SPECT/ceCT. The 30-day guideline time interval was exceeded in 62.5% of patients. The median relative tumour growth was higher in the population exceeding the guideline interval 8% (IQR: -1 to 28%) compared to those with an interval ≤ 30 days 6% (IQR: -6 to 11%) (Fig. 1) although this difference was not statistically significant (*p* = 0.30).

### Outcomes for the conventional and hybrid workflows

**Fig. 2:**
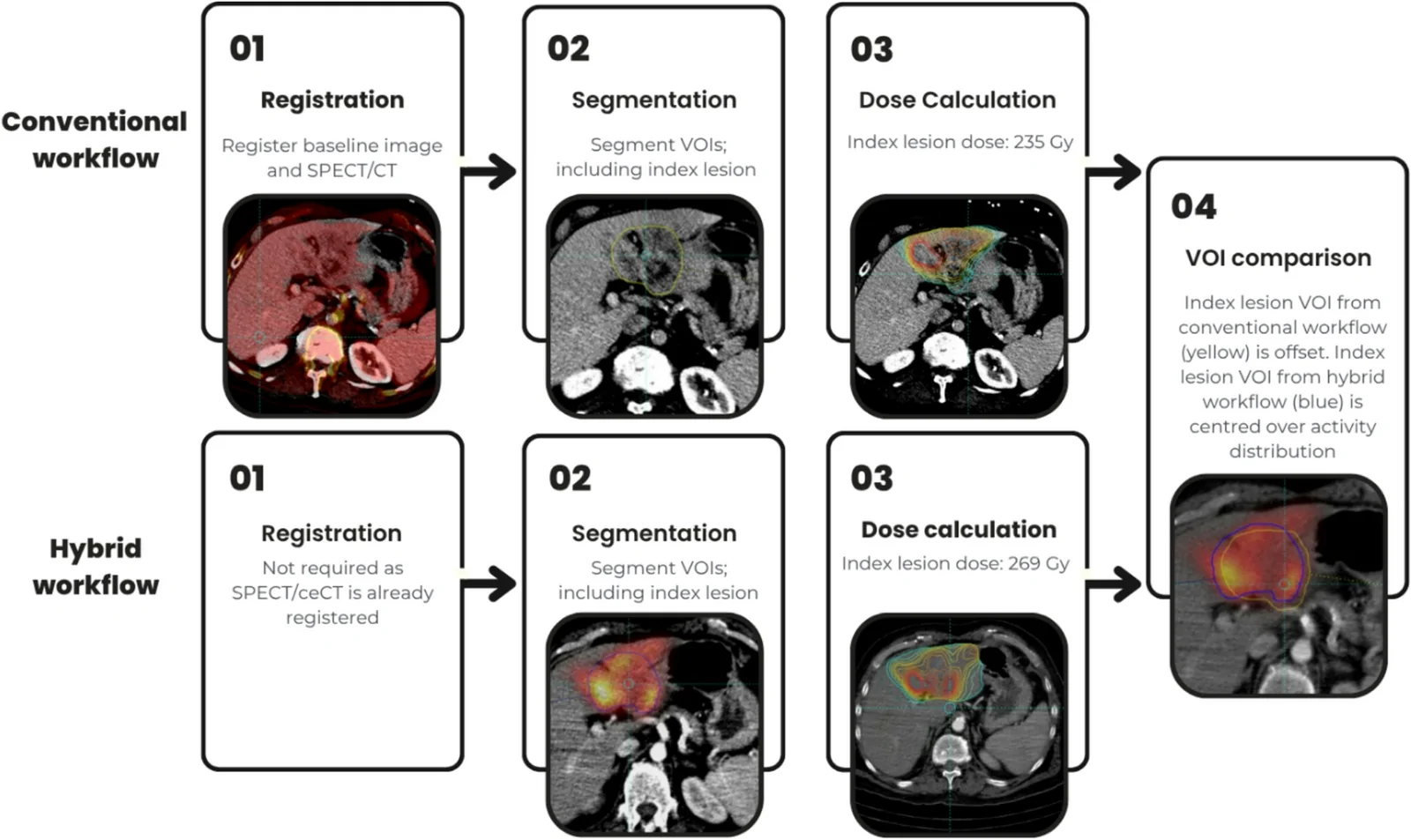
Radioembolization treatment planning steps for the conventional and hybrid workflow. Misalignment between the baseline CT and SPECT/CT is apparent in the conventional workflow, resulting in poor coregistration quality at the index lesion and incomplete capture of the [99mTc]Tc-MAA distribution. In the hybrid workflow, the SPECT/ceCT is inherently registered and the index lesion VOI is correctly centred over the count distribution

With the conventional workflow, the median D_index_ was 248.9 Gy (IQR: 198.6 to 394.8 Gy) with a median index lesion volume of 58.4 mL (IQR: 14.4 to 265.1 mL). The median D_n, perf_ was 84.7 Gy (IQR: 48.4 to 120.1 Gy) (Table 2). With the hybrid workflow, the median D_index_ was 271.1 Gy (range: 203.9 to 417.5 Gy) with a median index lesion volume of 73.2 mL (IQR: 20.9 to 315.2 mL) and the median D_n, perf_ was 78.9 Gy (range: 42.7 to 107.8 Gy). The median activity required to deliver a 205 Gy index lesion dose via the conventional workflow was 1.1 GBq (IQR: 0.5 to 2.6 GBq) and via the hybrid workflow was 0.9 GBq (IQR: 0.5 to 2.4 GBq), (Supplemental Table 1). No contrast nephropathy events were observed as part of the hybrid workflow as a consequence of the SPECT/ceCT procedure. Figure 2 illustrates a case example planned using both conventional and hybrid workflows.

### Dose comparison

**Fig. 3:**
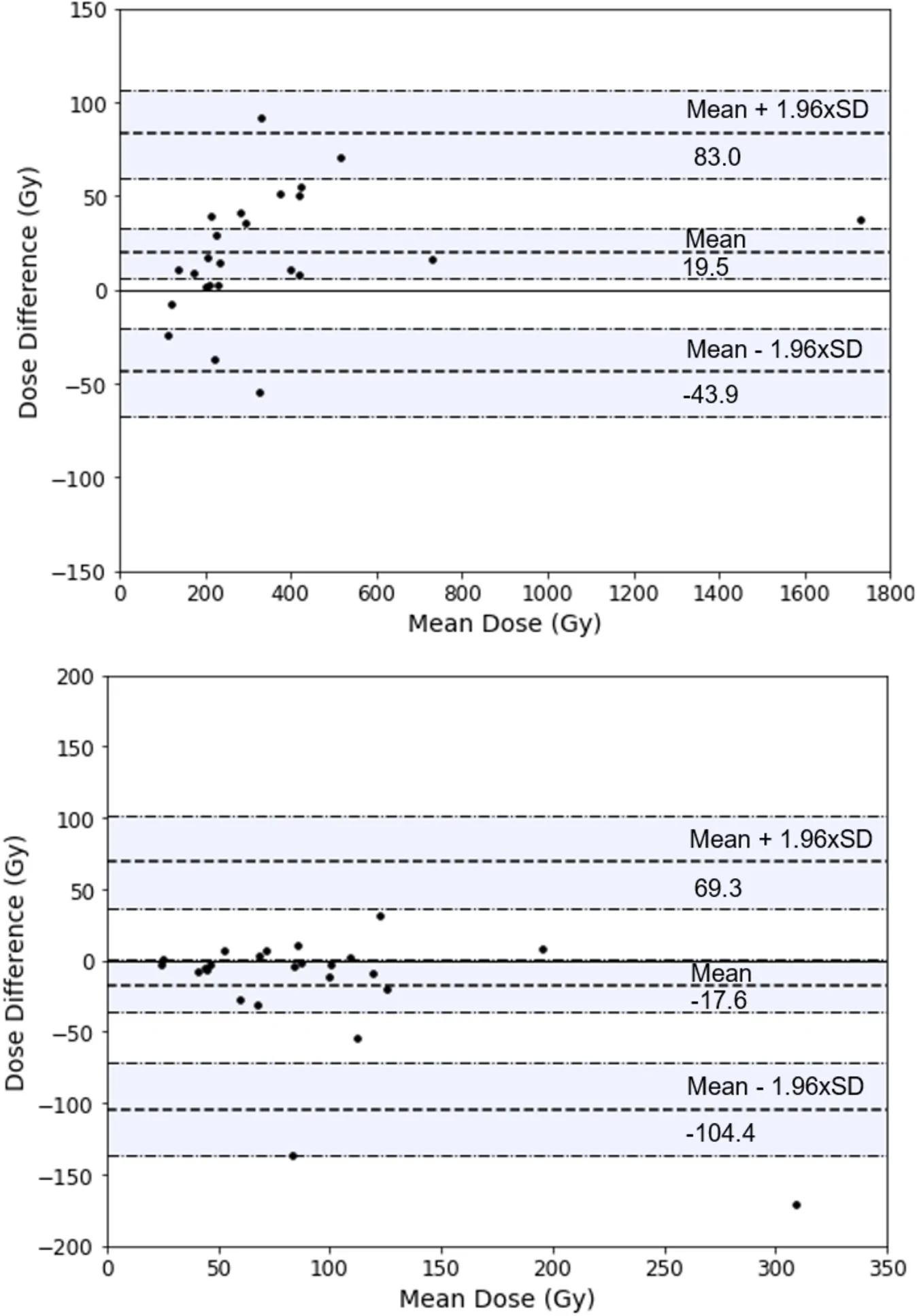
Bland-Altman plot for Index Lesion (top) and Perfused Volume Normal Tissue dose (bottom). The 95%limits of agreement are indicated by the bold dashed line and the surrounding shaded area representsthe 95% confidence interval about these limits

The Bland-Altman analysis of dose demonstrated that on average the hybrid workflow resulted in higher D_index_ (mean: 19.5 Gy, 95% limits of agreement [LoA]: -43.9 Gy, 83.0 Gy) (Fig. 2) and lower D_n, perf_ (mean: -17.6 Gy 95% LoA: -104.4 Gy, 69.3 Gy) versus the conventional workflow (Fig. 3). D_index_ (*p* = 0.008) differed significantly between workflows, but D_n, perf_ did not. The mean percentage difference in D_index_ between the two workflows was 5.6% (95% LoA: -17.1%, 28.3%), while the mean percentage difference in D_n, perf_ was − 10.7% (95% LoA: -58.2%, 36.5%) (Table 2).

**Table 2:** Dose and volume outcomes from the conventional and hybrid workflow

Parameter	Structure (Number of volumes)	Conventional Median (IQR) (Gy)	Hybrid Median (IQR) (Gy)	Mean Percentage Difference* (95% CI)	*P*-value
Dose	Index Lesion (24)	248.9 (198.6–394.8)	271.1 (203.9–417.5)	5.6 (-17.1–28.3)	**0.008**
	Perfused Volume Normal Tissue (24)	84.7 (48.4–120.1)	78.9 (42.7–107.8)	-10.9 (-58.2–36.5)	0.070
	Whole Liver Normal Tissue (24)	28.6 (18.8–40.9)	20.9 (13.4–31.1)	-17.3 (-66.3–31.7)	0.035
	Perfused Volume (24)	112.2 (68.7–149.0)	112.0 (76.8–149.6)	3.9 (-19.9–27.7)	0.859
Volume	Index Lesion (24)	58.4 (14.4–265.1)	73.2 (20.9–315.2)	13.5 (-55.9–82.9)	**0.014**

*The mean percentage difference is the average percentage change between protocols. Negative values indicate a reduction from the conventional to hybrid workflows

### Volume comparison

**Fig. 4:**
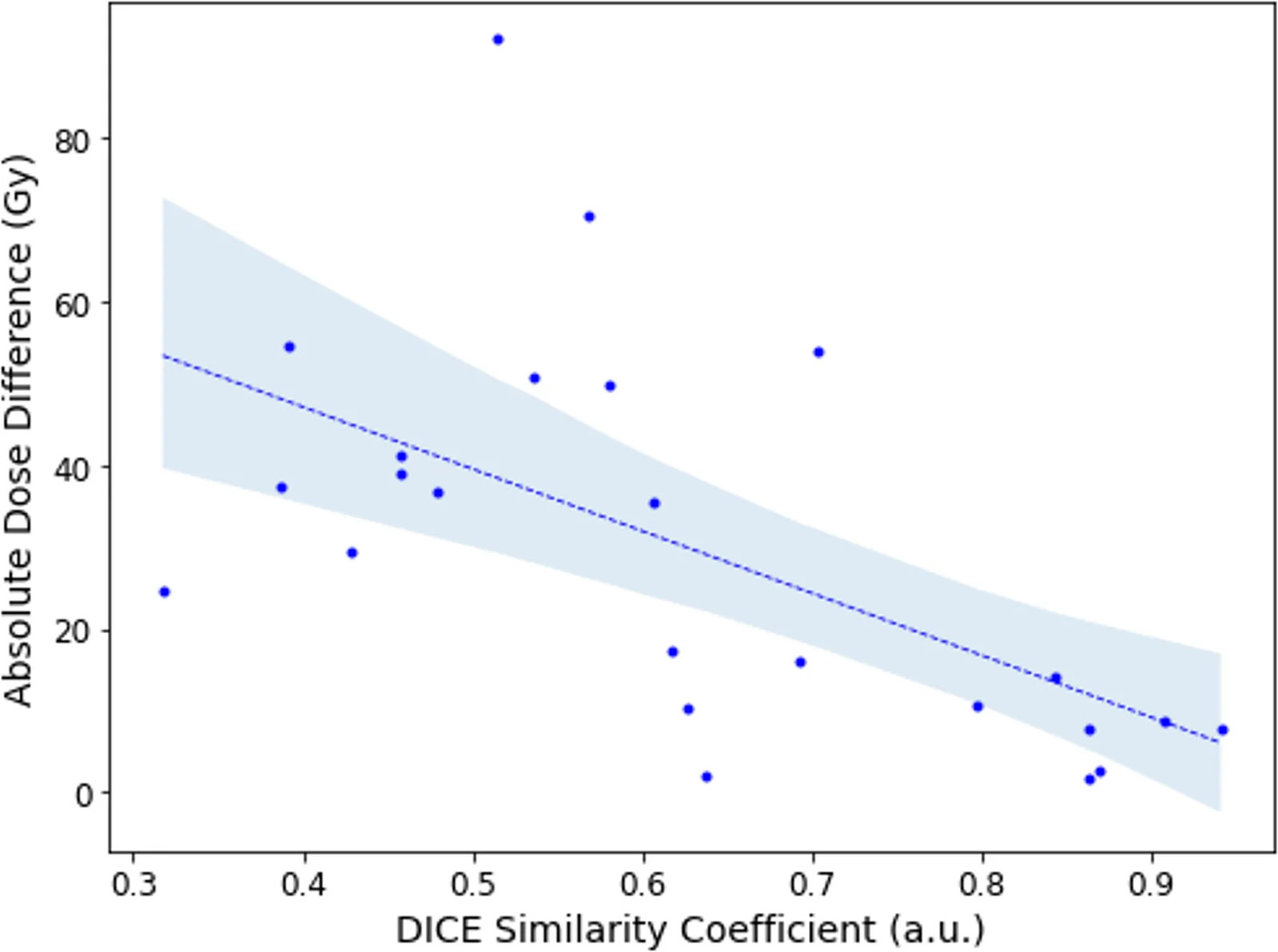
Linear regression plot for absolute dose difference against DICE similarity coefficient. The shaded area represents the 95% confidence interval

The mean percentage difference in index lesion volume (indicating interval progression) between the conventional and hybrid workflow was 13.5% (95% LoA: -55.9%, 82.9%). Index lesion volume was significantly greater for the hybrid workflow (*p* = 0.014). Contour intercomparisons were performed, yielding median values of 18.9 ± 12.6 mm for the Hausdorff distance, 4.9 ± 2.2 mm for the mean Distance to Agreement (mDTA), and 0.61 for the Dice Similarity Coefficient (DICE). DICE of < 0.5 was observed for 29% of patients, ≥ 0.5 to < 0.8 for 46% of patients and > 0.8 for 25% of patients. There was a significant linear correlation between DICE score and absolute dose difference (*p* = 0.02) (Fig. 4). A lower DICE score (indicating greater dissimilarity in index lesion contours) correlated with a larger discrepancy in dose, and as DICE score tended to one dose difference tended to zero.

### Clinical impact of hybrid SPECT/ceCT workflow

A statistically significant difference in index lesion volume was observed between the conventional (58.4 ml) and hybrid workflows (73.2 ml) *p* = 0.01. In 33% of patients, the index lesion volume increase relative to the conventional workflow was ≥ 10%. Similarly, a statistically significant difference in index lesion dose was noted between workflows (conventional: 248.9 Gy; hybrid: 271.1 Gy; *p* = 0.01), with 37.5% of patients experiencing a ≥ 10% increase in dose with the hybrid approach. Although the difference in perfused volume normal tissue dose between conventional and hybrid workflows (84.7 Gy vs. 78.9 Gy; *p* = 0.07) was not statistically significant, 41.7% of patients demonstrated a ≥ 10% reduction in this metric under the hybrid workflow.

## Discussion

The inclusion of an additional contrast-enhanced CT scan at the time of SPECT/CT imaging enables treatment planning based on hybrid SPECT/ceCT, which is theoretically advantageous by mitigating the risks of image misregistration and interval tumour progression. In this study, treatment planning using SPECT/ceCT was feasible and yielded significantly different index lesion volume and dose estimates, with a 13.5% increase in index lesion volume (*p* = 0.01) and a 5.6% increase in index lesion absorbed dose (*p* = 0.01) compared to the conventional workflow.

The challenges arising from conducting baseline imaging and SPECT/CT at different time points is discussed in several studies [25, 26] which outline that inaccurate CT and SPECT image registration significantly impacts the accuracy of quantitative activity estimation and tumour to normal tissue (T: N ratio) assessment. The primary contributing factor being inaccurate VOI segmentation based on a mismatched CT. A recommendation from Lyu et al. [25] therefore was to perform registration of images captured at the same time point to ensure dosimetric accuracy.

Index lesion dose differed significantly between the considered workflows, with doses being higher in the hybrid workflow where the need for co-registration is removed. An increase in index lesion dose between workflows may be expected if the index lesion volume is reduced (i.e., the dose is averaged over a smaller mass) or if a greater proportion of counts are captured within the index lesion VOI. In this study, the observed dose increase is most likely attributable to improved image co-registration and consequently, more accurate count attribution within the index lesion VOI, particularly given that the index lesion volume was on average greater in the hybrid workflow. Although the difference in perfused volume normal tissue dose between conventional and hybrid workflows (84.7 Gy vs. 78.9 Gy; *p* = 0.07) was not statistically significant, doses were lower on average in the hybrid workflow.

The median time interval between baseline imaging (CT/MRI) and SPECT/CT was 5.9 weeks, which represents a reasonable time frame that may be expected in clinical practice based on previous studies [4, 9]. The aggressiveness of tumour growth varies among patients, and it is challenging to predict individual disease progression rates. Consequently, a maximum interval of 30 days is suggested in guidelines to minimize opportunity for interval progression. Results indicated that the subgroup of patients with an interval exceeding the 30-day guideline exhibited greater tumour growth than those with ≤ 30 day interval, although this difference was not statistically significant. Implementing SPECT/ceCT renders the question of optimal time interval moot as the ‘interval’ is eliminated. By designing the treatment plan based on the most up to date information any potential interval progression is captured and accounted for.

The difference in alignment between the index lesion in the registered baseline image and SPECT/ceCT (as indicated by the mDTA) was in line with differences reported previously for separate modality scanners. Nakamoto et al. [26] reported that the deviation in static lesions below the diaphragm between registered PET/CT and ceCT was 6.9 ± 2.9 mm, which indicates that the median discrepancy in this study 4.9 ± 2.2 mm is a reasonable difference to expect when registering separate images. A DICE coefficient for index lesions in the respective images was also determined and a score of < 0.8 was categorised as poor. The majority of patients (75%) had a DICE score < 0.8, indicating generally poor coregistration and low structure overlap at the index lesion. It was evident that as DICE tended to one (indicating complete overlap of the index lesion in the two workflows) dose difference tended to zero, in other words for structures that agreed between workflows the same dose was output. In practice, no patients had a DICE of 1, and in a substantial percentage of patients (33%) the index lesion volume change on the SPECT/ceCT relative to the baseline CT was ≥ 10%.

From a clinical perspective, key parameters from the treatment plan include tumour and normal tissue absorbed dose (as indicators of efficacy and safety), and tumour volume measurements (for use in response evaluation). A workflow that ensures accurate tumour and normal liver dose determination and tumour volume measurement may consequently be highly impactful. There are also a number of additional benefits that arise from incorporating contrast-enhanced CT into the SPECT/CT procedure, including a more streamlined diagnostic assessment and treatment planning process. However, whilst the advantages offered by this workflow (namely improvement in VOI boundary definition) are beneficial, if downstream dose differences are dominated by uncertainties arising from inter-operator segmentation variability [27], then this workflow is only the first step towards improved accuracy and the clinical relevance should be interpreted in the broader uncertainty framework.

Since SPECT/ceCT scanning requires a SPECT scanner equipped with helical CT and availability of an intravenous contrast injector, one of the barriers preventing routine adoption of this technique could be lack of access to adequate equipment. First generation SPECT/CT devices that are equipped only with a low-dose CT component do not offer the flexibility of contrast enhanced imaging protocols. SPECT/CT procedures are typically conducted in nuclear medicine departments where contrast is rarely used – a 2012 global survey of nuclear medicine departments indicated that only 4% of responding sites had access to a dedicated intravenous contrast injector for all SPECT/CT systems [28]. For sites that have access to the necessary equipment, resource management is still a consideration – if by addition of a contrast enhanced CT a fully integrated SPECT/CT report including cross verification is required, an increase in the average reporting time may be expected. Nuclear medicine staff must also be sufficiently trained in the use of intravenous CT contrast agents. Finally, bilinear scaling methods utilised in CT attenuation correction of SPECT data are complicated in a ceCT setting, as they fail to adequately scale contrast enhanced tissues [29, 30]. For this reason, our centre performs attenuation correction using the low dose CT component. However, it has been reported that while attenuation correction with ceCT may give rise to artefacts, there is a minor influence on calculated attenuation coefficients [31].

The power of this study is limited by the small population size considered; results should be interpreted in this light. There was little variation in the anatomical distribution of lesions (21 out of 24 patients had lesions located in the right liver lobe), which may suggest a potential selection bias, however there is no reason to believe that an alternative lesion distribution would be impacted differently by the presence of intravenous contrast. The 2.5 cm size threshold excludes smaller lesions potentially amenable to radioembolization but avoids increased dosimetric variability from pronounced partial volume effects at smaller sizes. This cutoff, derived from phantom-based evaluations, preserves contrast recovery while maximizing the number of lesions suitable for reliable analysis. The contour comparison metrics considered provide only a gross measure of difference and lack the specificity required to definitively comment on whether the origin of dosimetric differences primarily result from misregistration, tumour progression or other factors, including time-separated segmentations or inter-observer variability effects. Finally a limitation of this study is that it retrospectively assessed differences in dosimetric metrics rather than direct clinical outcomes. The evaluation of clinical value requires prospective studies with appropriate clinical endpoints.

SPECT/ceCT scanning is an underutilized technique in the context of radioembolization treatment planning. However, its adoption may increase as more nuclear medicine departments incorporate contrast-enhanced CT scanning into their procedures. This integration is already evident in diagnostic studies, such as ^111^In Octreotide SPECT [31]. Before the widespread implementation of SPECT/ceCT-based treatment planning, consideration should be given to breathing protocols and the best methods to match respiratory states during SPECT and ceCT [32, 33]. Respiratory gating is available across several PET/CT vendors [34, 35], and has been demonstrated to reduce and quantify organ motion [36], as well as improve visualisation of small lesions [37], however, it is not yet routinely implemented in clinical practice. Respiratory gating in SPECT/CT may prove beneficial, but further evaluation of its impact on clinical practice is required.

## Conclusion

Hybrid [99mTc]Tc-MAA SPECT/ceCT treatment planning is technically feasible and leads to significant differences in dosimetry metrics compared with the conventional method.

## Electronic Supplementary Material

Below is the link to the electronic supplementary material.

Supplementary Material 1

## Data Availability

The datasets used and/or analysed during the current study are available from corresponding author on reasonable request.

## Abbreviations

ceCT: contrast enhanced computed tomography
SPECT/CT: single photon emission computed tomography/computed tomography
ldCT: low dose computed tomography
HCC: hepatocellular carcinoma
LoA: limits of agreement
^99m^Tc-MAA: Technetium-99 macroaggregated albumin
MCD: multi-compartment dosimetry
MIRD: medical internal radiation dose
MRI: magnetic resonance imaging
eGFR: estimated glomerular filtration rate
OSEM: ordered subset expectation maximisation
LSF: lung shunt fraction
VOI: volume of interest
AC: attenuation correction
T:N: tumour to normal tissue ratio
D_whole_: Dose to whole liver tissue
D_perf_: Dose to perfused volume
D_n,perf_: Dose to perfused volume normal tissue
D_n,whole_: Dose to whole liver normal tissue
D_index_: Dose to index lesion
HD: Hausdorff distance
mDTA: mean distance to agreement
