# Supplementary material for: A novel approach to radioembolization treatment planning using a hybrid [99mTc]Tc-MAA contrast enhanced SPECT/CT
Source: Eur J Nucl Med Mol Imaging. 2026 Jul 11;53(12):7038–48. doi: 10.1007/s00259-026-08027-6 (PMC13633308; doi:10.1007/s00259-026-08027-6)
Supplement: Supplementary file 1 [file 259_2026_8027_MOESM1_ESM.docx]

# SUPPLEMENTAL TABLES

**Table 1. Activity required to deliver an index lesion dose of 205 Gy calculated via the conventional and hybrid workflow**

| *Patient ID* | *Activity required to deliver a D_index_ of 205Gy*  *(conventional workflow)*  *(GBq)* | *Activity required to deliver a D_index_ of 205Gy*  *(hybrid workflow)*  *(GBq)* | *Difference in activities**  *(GBq)* |
| --- | --- | --- | --- |
| 1 | 2.27 | 1.98 | 0.29 |
| 2 | 4.17 | 4.45 | 0.28 |
| 3 | 0.36 | 0.31 | 0.05 |
| 4 | 2.17 | 1.64 | 0.53 |
| 5 | 0.32 | 0.27 | 0.04 |
| 6 | 0.89 | 0.87 | 0.02 |
| 7 | 1.66 | 1.46 | 0.21 |
| 8 | 0.82 | 0.97 | 0.15 |
| 9 | 0.82 | 0.72 | 0.09 |
| 10 | 0.49 | 0.61 | 0.12 |
| 11 | 2.67 | 2.22 | 0.45 |
| 12 | 0.42 | 0.37 | 0.05 |
| 13 | 2.79 | 2.58 | 0.21 |
| 14 | 0.52 | 0.48 | 0.04 |
| 15 | 0.94 | 0.92 | 0.02 |
| 16 | 3.81 | 3.76 | 0.05 |
| 17 | 4.86 | 4.62 | 0.24 |
| 18 | 1.52 | 1.50 | 0.01 |
| 19 | 2.90 | 3.42 | 0.52 |
| 20 | 1.19 | 0.75 | 0.43 |
| 21 | 0.27 | 0.26 | 0.01 |
| 22 | 0.64 | 0.62 | 0.01 |
| 23 | 0.33 | 0.29 | 0.04 |
| 24 | 2.48 | 2.46 | 0.02 |

*Absolute activity difference between conventional and hybrid workflows
